# Effects of different light incident angles via a head-mounted device on the magnitude of nocturnal melatonin suppression in healthy young subjects

**DOI:** 10.1007/s41105-021-00360-7

**Published:** 2022-01-21

**Authors:** Naoko Kubota, Yusuke Tamori, Kenkichi Baba, Yujiro Yamanaka

**Affiliations:** 1 Laboratory of Life and Health Sciences, Faculty of Education and Graduate School of Education, Hokkaido University, Sapporo, 060-0811 Japan; 2 Department of Nursing, Hokkaido University of Science, Sapporo, Japan; 3 DENSEI COMTEC Inc., Ebetsu, Japan; 4 Department of Pharmacology and Toxicology, Neuroscience Institute, Morehouse School of Medicine, Atlanta, GA USA; 5 Research and Education Center for Brain Science, Hokkaido University, Sapporo, Japan

**Keywords:** Head-mounted light devices, Bright light therapy, Melatonin suppression, Pupil constriction, Humans

## Abstract

Bright light is a primary zeitgeber (synchronizer) for the central circadian pacemaker in humans. Recently, head-mounted devices for light therapy have been developed to treat patients suffering from circadian rhythm sleep disorders. In this study, to evaluate the influence of the light incident angle of head-mounted devices on the human circadian pacemaker, we examined the effects of bright light (ca.10000 lx) from two different angles (55° vs. 28°) on the suppression of melatonin secretion at night. Twenty-nine subjects (25.1 ± 6.3 SD years) participated in the present study. The subjects were kept under dim light conditions (< 5 lx) from 4 h before their habitual bedtime, followed by exposure to 1 h of bright light at two different angles during their habitual bedtime. Saliva samples were collected every hour under dim light conditions and then collected every 30 min during the bright light exposure. To assess the effect of the light incident angle on ipRGCs mediating light-evoked pupillary constriction, pupil size was measured in before and after exposure to bright light. Melatonin suppression in the group exposed to light at 28° was significantly higher than that in the group with light at 55° (*p* < 0.001). The pupillary constriction was significantly greater in the group exposed to light at 28° than that in the group with light at 55° (*p* < 0.001). The present findings suggest that the light incident angle is an important factor for bright light therapy and should be considered to effectively use head-mounted devices in home and clinical settings.

## Introduction

Bright light is a primary zeitgeber (synchronizer) for the circadian pacemakers in mammals, including humans. The central circadian pacemaker is located in the suprachiasmatic nucleus (SCN), which directly receives environmental light information from the eye via the retinohypothalamic tract [1]. Under constant environment conditions in the temporal isolation facility, free-running circadian rhythms of core body temperature and sleep–wake cycle have a period of 25.0 h on average [2]. The free-running rhythms of the rectal temperature and the sleep–wake cycle are entrained by timed exposures to bright light [3]. Failure of this synchronization may influence many pathologies [4]. Therapeutic timed light exposure has been established as a treatment for depression [5], seasonal affective disorder [6], and circadian sleep phase disorders [7]. Bright light therapy often uses a bright light box that emits bright light up to 10,000 lx at the recommended distance and duration. Recently, head-mounted devices have been developed and are easily used for the bright light therapy in clinic and home. The light devices are equipped with light-emitting diodes (LEDs) with short-wavelength light (blue-green colors), which is particularly effective in melanopsin-expressing intrinsically photosensitive retinal ganglion cells (ipRGCs) [8].

Light exposure via a head-mounted device suppresses the level of melatonin concentration at night [9], and timed exposure successfully phase shifts the melatonin onset [9–11] which can be used as the marker of the circadian pacemaker in the SCN. Additionally, timed bright light exposure via a head-mounted device improves the quality of night sleep in adolescents affected by delayed sleep phase syndrome [12], improves the wakefulness/mood over the morning hours, and decrease night awakenings [13]. Although, several kinds of light therapy devices are available, the position and angle of device light varies and has not been considered. According to earlier studies of human visual field perception and ergonomics, an angle of 55°–60° from the horizontal visual site is the upper limit of visual field, and an angle of 30° is considered as the threshold of central visual perception [14, 15].

In the present study, we investigated the effect of the light incident angle on nocturnal melatonin production in healthy subjects. We also measured pupil diameters for the indication of amount of light received by the subject’s retina while they wore the devices.

## Materials and methods

### Subjects

A total of 29 (20 male and nine female) subjects aged 19–44 years (mean ± standard deviation, 25.1 ± 6.3 years) participated in the present study. None of the subjects had to work early in the morning, late at night, or in rotating night shifts. None of the subjects had a history of eye disease, psychiatric, endocrine, or sleep disorders. All subjects provided written informed consent before entering the study, and they were able to withdraw from the experiment whenever they wanted. This study was approved by the ethics committee of the Hokkaido University Graduate School of Education (no.17–47) and was conducted according to the Declaration of Helsinki.

### Melatonin suppression protocol

**Fig. 1:**
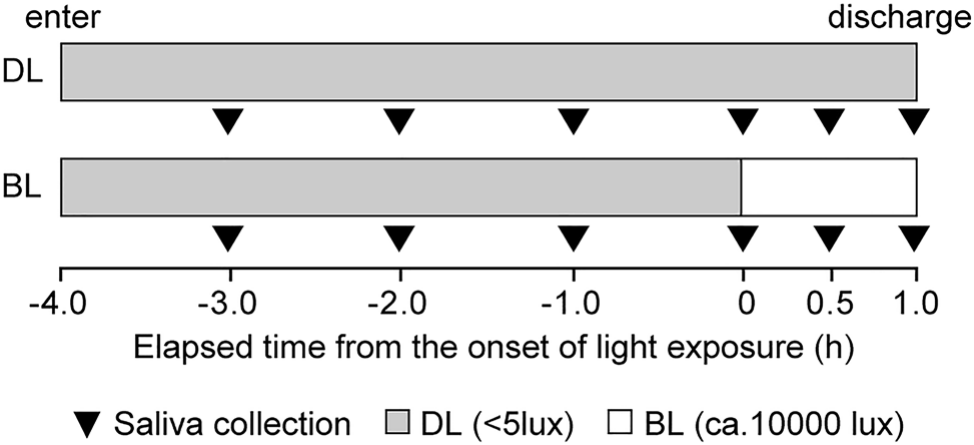
Experimental protocol of melatonin suppression test. Experimental protocol for the laboratory-controlled assessment of the melatonin concentration under dim light conditions (DL) and melatonin suppression by 1-h bright light (BL) from the angle of 55°or 28°

A total of 22 (15 male and 7 female) subjects aged 19–38 years (mean ± standard deviation, 23.9 ± 4.3 years) were studied in the melatonin suppression test. In the present study, a threshold of melatonin concentration before the exposure to bright light was 4 pg/ml [16]. One subject was excluded from the data analysis due to the low salivary melatonin concentrations. The experimental protocols are illustrated in Fig. 1. Beginning 2 weeks before the experiment, the subjects were instructed to keep their regular sleep–wake cycle and to wear a data collection device (MotionWatch 8, CamNtech, UK; Actiwatch-L, Minimitter, USA) that recorded wrist activity and light intensity. The subjects came to the laboratory 4 h before bedtime. The light intensity in the laboratory was less than 5 lx at eye level, which was expected to not affect nocturnal melatonin secretion until the time of bright light exposure for 1 h. The subjects were instructed to sit on a chair throughout the experiment. The experiments consisted of two different light conditions (dim light and bright light) with a randomized cross-over designed, which were conducted at 1-week intervals. In the bright light condition, the subjects were instructed to wear a head-mounted bright light device for 1 h from their habitual bedtime. Saliva samples were collected using a cotton swab (Sarstedt, Nümbrecht, Germany) every 1 h until the subject’s habitual bedtime and every 30 min during the 1-h bright light exposure (Fig. 1). Immediately after saliva sampling, the cotton swab was inserted into a collection tube, and the tube was centrifuged (20 min at 2000 rpm). The saliva samples were stored at − 30 °C until the melatonin assay was performed.

### Bright light exposure with the head-mounted device

**Fig. 2:**
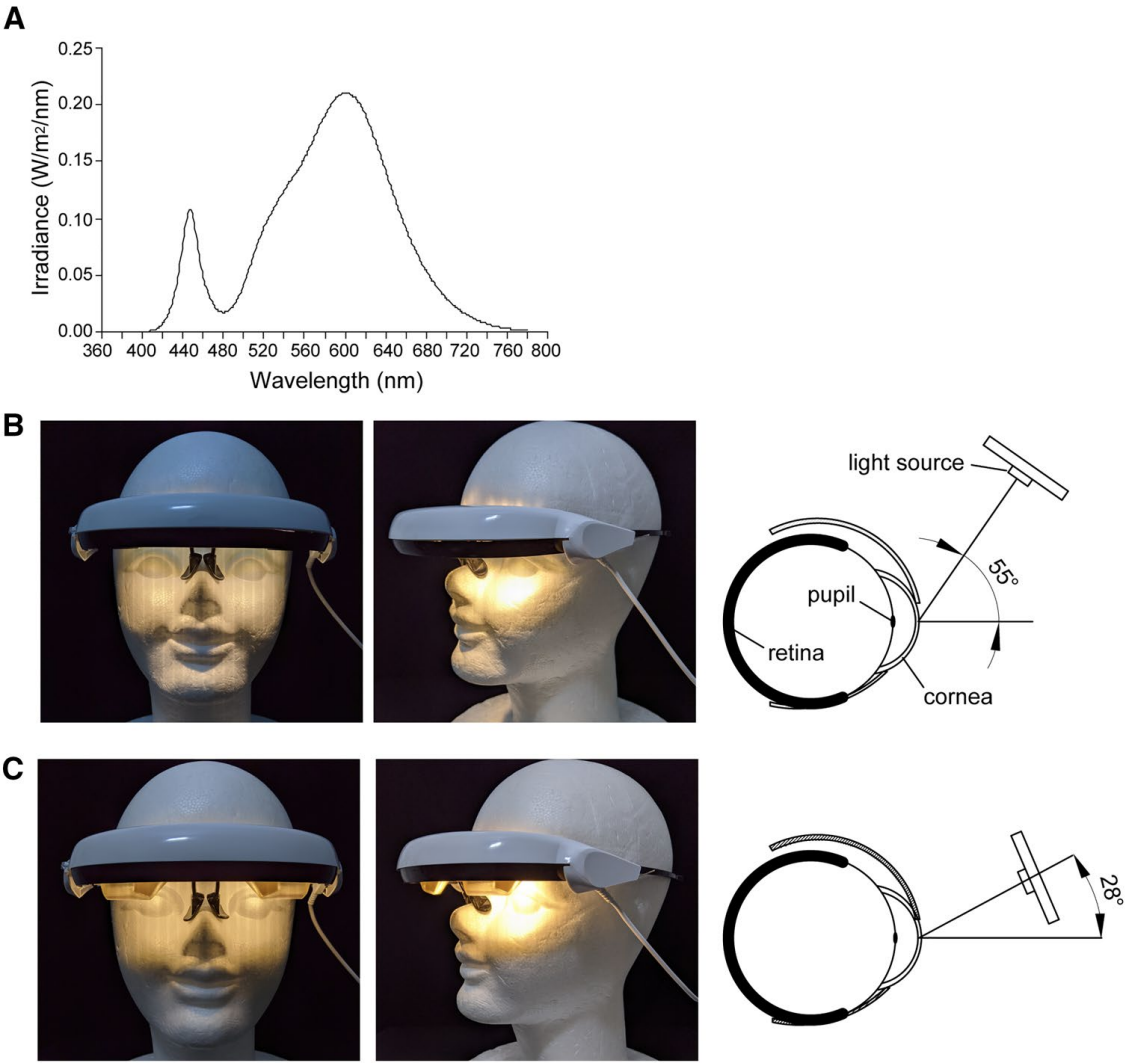
Spectrum profile of the light-emitting diodes (LEDs) and light incident angles of the head-mounted light device. Spectrum profile of the LEDs (**A**), images and schematic model of light incident angles at 55° (**B**) and 28° (**C**) of the head-mounted light devices used in the present study

The subjects were instructed to wear a custom-made head-mounted light device (glasses type, Fig. 2) (DENSEI COMTEC Inc., Japan) during the 1-h bright light exposure period. The spectral distribution of the light source (Fig. 2A) and the light incident angle of the head-mounted devices are illustrated (Fig. 2B, C). The wavelength of the LEDs was set at 2963 K. To assess the effect of the light incident angle on nocturnal melatonin secretion, this angle was set at 55° (Experiment 1: bright light at 55°, Fig. 2B) or 28° (Experiment 2: bright light at 28°, Fig. 2C). The subjects were instructed to stay awake, while sitting on a chair during the 1-h bright light exposure.

### Estimation of light influx of cornea and retina

To evaluate the light intensity at the level of cornea and retina from the head-mounted light device, we developed a custom-made human eye model using two acrylic hemispherical domes (25.4 mm in diameter) [17] which are similar to the average human eye size. In the front of eye model, a 2 mm hole was made as the estimated pupil size and a 12 mm hole was made at the back of eye model for measuring light intensity of the central retina. The corneal and retinal light intensities were measured using an illuminance spectrophotometer (CL-500A, Konica Minolta, Japan). The value of light intensities was calculated from five measurements for each light angle at 55° or 28°.

### Salivary melatonin assay

Salivary melatonin concentrations were measured using a direct saliva melatonin radioimmunoassay kit (kit No. RK-DSM2-U, Bühlmann Laboratories AG, Switzerland) according to the manufacturer’s instructions. The detection threshold of the assay was 0.5 pg/ml. The inter-assay and intra-assay variations were 10.0% and 11.3%, respectively. The percentage of light-induced melatonin suppression was calculated as follows: ([melatonin concentration under dim light at 1.0 h − melatonin concentration under bright light at 1.0 h]/melatonin concentration under dim light at 1.0 h) × 100.

### Measurement of pupil constriction by exposure to a bright light

To assess the effect of the light incident angle on ipRGCs mediating light-evoked pupillary constriction, seven (three male and four female) subjects aged 21–44 years (mean ± standard deviation, 23.9 ± 4.3 years) were studied in the measurement of pupil size in darkness and after exposure to bright light at two different light angles. The subjects came to the laboratory at 16:00–17:00 h. At the start of the experiment, the subjects wore the head-mounted bright light device and a custom-made head-mounted video camera (Takei Scientific Instruments Co., Ltd., Japan) equipped with an infrared LED, which does not affect the pupil size. The measurement of pupil size in darkness was performed at least 5 min until the pupil diameter no longer increased. Afterward, the head-mounted bright light device was turned on, and measurement of the pupil diameter continued for 5 min. This procedure was repeated again to measure the different light angle with counterbalancing. All the data were stored in a personal computer. The pupil diameters were estimated based on the size of the horizontal pupil diameter. The pupil constriction was expressed as a percentage of the pupil diameter in the darkness prior to the bright light exposure.

### Statistical analyses

Data are expressed as the mean ± standard error of the mean. Analyses of time series data were performed using the two-way repeated measures analysis of variance (ANOVA) with the post hoc Bonferroni adjusted significance tests. Comparisons of two independent or dependent values were analyzed using the unpaired *t* test or paired *t* test, respectively. GraphPad Prism version 8 (GraphPad Software Inc., CA, USA) was used for all statistical analyses. A *p* value < 0.05 was considered a statistically significant difference.

## Results

### Illuminance measurement

Table 1 summarizes the light intensities at the cornea and retina measured using the human eye model. Our results show the angles of light did not alter the light intensity at the level of cornea (ca. 10,000 lx), but the intensity of the central retina was three times higher with the light angle at 28° (231 lx) than the light angle at 55° (75 lx).

**Table 1:** Light intensity at center of cornea and retina

Angle	Cornea		Retina	
	Lux	W/m^2^	Lux	W/m^2^
55°	10,386	30.62	75	0.23
28°	9999	28.94	231	0.65

The light intensities are measured with photometer 5 times by using a custom-made human eye model. Values are expressed as the averaged light intensity in Lux and W/m^2^, respectively

### Melatonin suppression

**Fig. 3:**
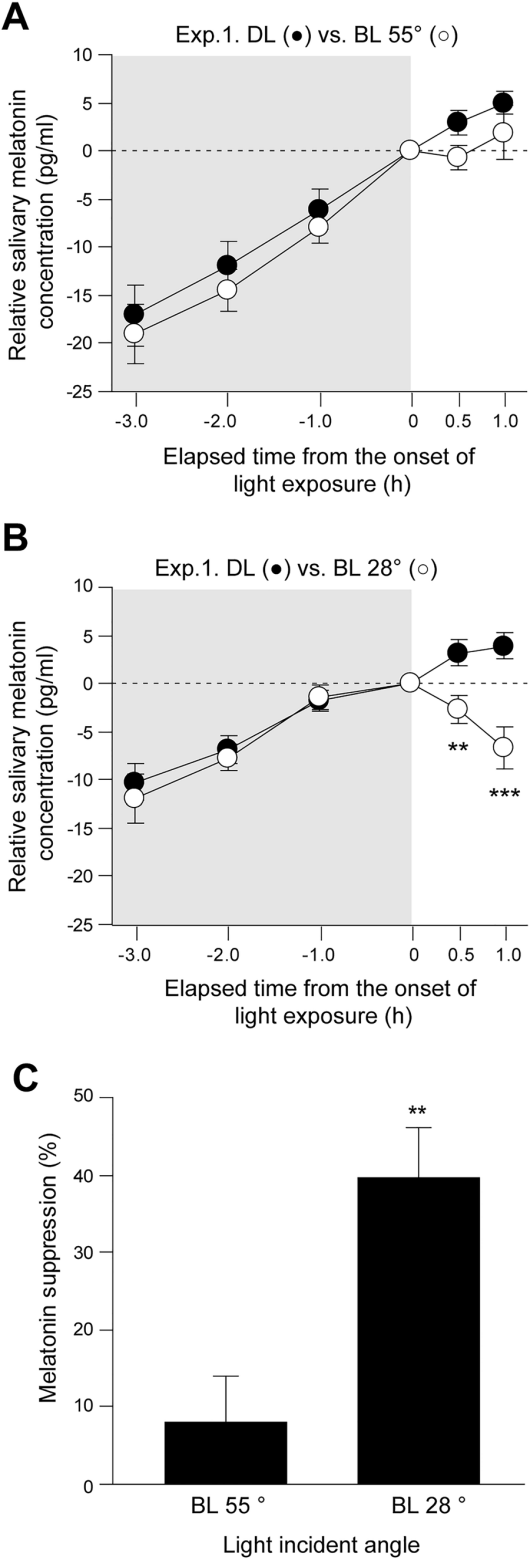
Salivary melatonin concentrations under dim light and bright light at different angle and melatonin suppression. Mean salivary melatonin concentrations measured in Experiment 1 [dim light (closed circles) vs. light angle of 55° (BL55°, open circles)] (**A**) and Experiment 2 [dim light (closed circles) vs. light angle of 28° (BL28°, open circles)] (**B**). The zero value corresponds to the melatonin concentration at 0 h just before the bright light exposure. Gray and white areas indicate the time of dim and bright light, respectively (**p = 0.007, ***p < 0.001, two-way repeated measures ANOVA with post hoc Bonferroni adjusted test). The percentage of melatonin suppression (**C**) by light at 28° (BL28°) was significantly larger than that at 55°(BL55°) (**p = 0.003, unpaired *t* test). The percentage of melatonin suppression was calculated as follows: ([melatonin concentration under dim light at 1.0 h − melatonin concentration under bright light at 1.0 h]/melatonin concentration under dim light at 1.0 h) × 100

The mean salivary melatonin concentration under dim light and bright light conditions and percentage of melatonin suppressions are shown in Fig. 3. The melatonin concentrations were normalized, setting the concentrations measured at 0 h as zero, because the melatonin concentrations showed an inter-individual difference. In subjects exposed to a light incident angle of 55°, the two-way repeated measures ANOVA test revealed no significant interaction between the time points (− 3.0, − 2.0, − 1.0, 0, 0.5, and 1.0 h) and light conditions (dim light vs. light at an angle of 55°; *p* = 0.08, Fig. 3A), although the melatonin concentrations were slightly lower when subjects were exposed to light at an angle of 55° compared to dim light conditions (dim light vs. light angle at 55°: 2.9 ± 1.3 pg/ml vs. − 0.7 ± 1.3 pg/ml at 0.5 h, 5.0 ± 1.1 pg/ml vs. 1.9 ± 2.8 pg/ml at 1.0 h). In contrast to the light angle at 55°, the melatonin concentrations in subjects exposed to light at an incident angle of 28° showed significant interactions in the two-way repeated measures ANOVA test (*p* < 0.001). The post hoc Bonferroni adjusted test revealed that the melatonin concentrations in subjects exposed to bright light at a 28° angle were significantly lower than in subjects exposed to dim light conditions (dim light vs. bright light at an angle of 28°: 3.1 ± 1.3 pg/ml vs. − 2.8 ± 1.5 pg/ml at 0.5 h, *p* = 0.007; 3.9 ± 1.3 pg/ml vs. − 6.7 ± 2.2 pg/ml at 1.0 h, *p* < 0.001; Fig. 3B). The percentage of melatonin suppression by light at an angle of 55° was significantly lower than that by light at an angle of 28° (light angle of 55° vs. 28°: 8.0 ± 6.1% vs. 39.7 ± 6.4%, respectively, *p* = 0.003, unpaired *t* test; Fig. 3C).

### Pupillary constriction

**Fig. 4:**
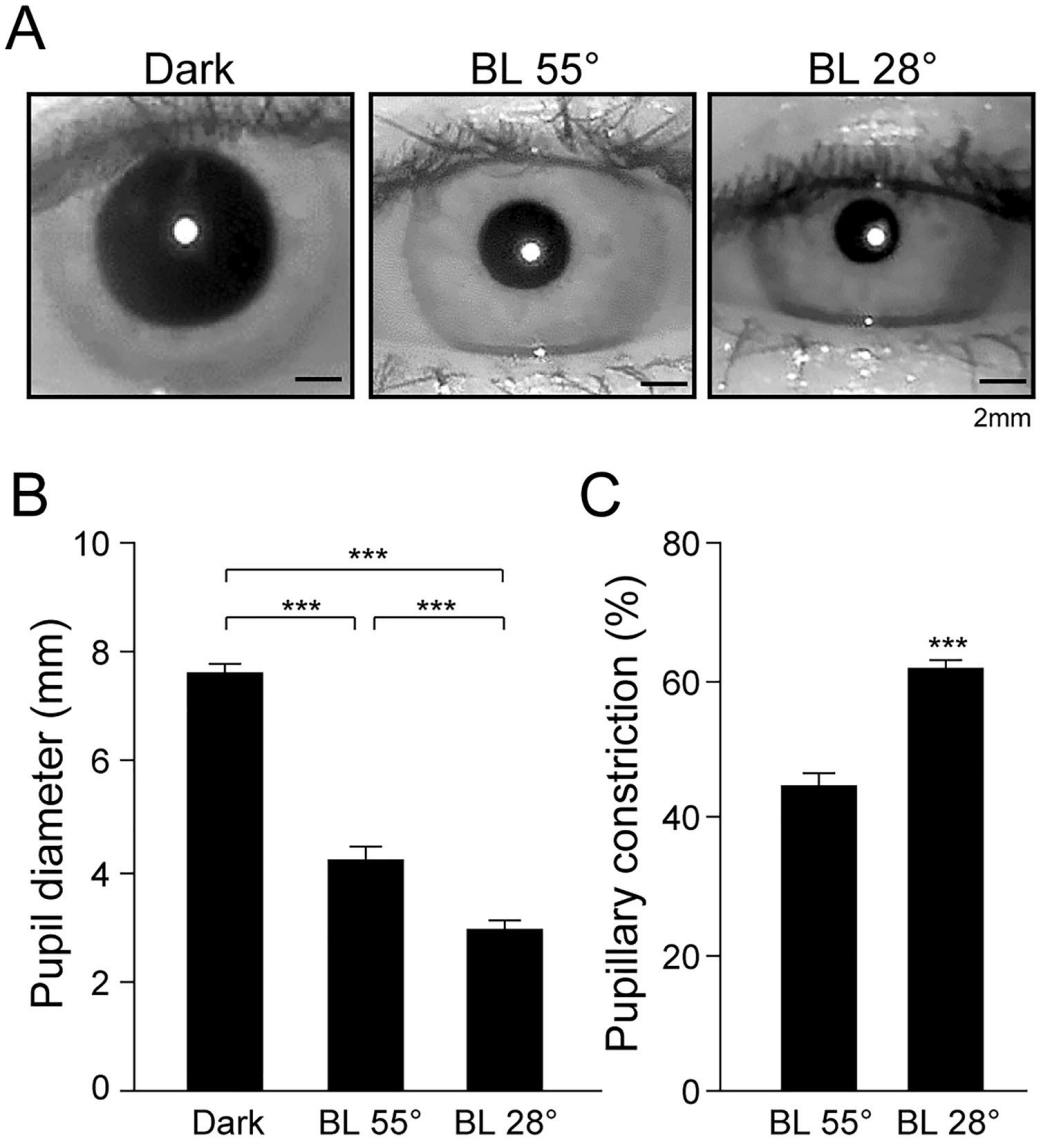
Pupil diameter and pupillary constriction in response to different light angles. Representative pupil images showed pupillary constriction in response to 5 min bright light at of 55° (BL55°) and 28° (BL28°) in the same subjects (**A**). The mean pupil diameters (**B**) and pupillary constriction in response to different light angles (**C**). Asterisks indicate the significant difference in each conditions (***p < 0.001, Bonferroni adjusted test or paired *t*-test)

Figure 4 indicates representative pupil images measured in dark and during the exposure to bright light at an angle of 55° and 28° (Fig. 4A), mean pupil diameter (Fig. 4B) and pupillary constriction (Fig. 4C). Repeated-measures ANOVA with post hoc Bonferroni adjusted test revealed the significant changes in the pupil diameters in dark and light at an angle of 55° and 28° (*p* < 0.001). The mean pupil diameter in the dark was 7.6 ± 0.5 mm. After exposure to light, the pupil diameters were significantly constricted by light at two light angles (4.2 ± 0.7 mm in light at 55°, 2.9 ± 0.4 mm in light at 28°, Fig. 4B). The percentages of pupillary constrictions were significantly larger in the light at 28° (61.6 ± 4.2%) than in the light at 55° (44.7 ± 6.6%) (*p* < 0.001, paired *t* test, Fig. 4C).

## Discussion

The present findings suggest that subjects exposed to bright light during the first hour of their habitual bedtime were influenced by the angle of light incidence. The subjects exposed to bright light at an incident angle of 28° showed significantly higher melatonin suppression than subjects exposed to light at an angle of 55° (Fig. 3). The magnitude of melatonin suppression is influenced by the light intensity [18] and the range of wavelength [9]. We estimated the luminous flux received by the central retina using the human eye model equipped with a luminometer probe and our observation indicates the more light was received in the central retina when light was exposed from lower angle (angle of 28°) than the higher angle of light (angle of 55°) (Table 1). The spectrum profile of LEDs used in our device also showed a peak at the short wave range where the suppression of nocturnal melatonin production is most occurred [9]. These evidence support, with the right angle, the light source we used in the study were sufficient to suppress melatonin secretion.

Melanopsin is the nonvisual photopigment coded by OPN4 gene expressed in ipRGC and melanopsin signaling plays an important role in circadian regulation and pupillary light reflex [19, 20]. Melanopsin-positive ipRGC is most sensitive for blue light [21] very similar to the action spectrum of acute nocturnal melatonin suppression [9]. The recent study also suggests the pineal melatonin synthesis is predominantly driven by melanopsin signaling in human [22]. Melanopsin-positive M1 ipRGC neurons directly project to the SCN and olivary pretectal nucleus (OPN) which controls pupillary reflex [23]. Both signals are then relayed to the superior cervical ganglion, a part of autonomic nervous system, and those signals are individually projected to the pupillary dilator muscle and pineal gland to regulate melatonin synthesis [24, 25]. Our results from both melatonin suppression and pupillary constrictions possibly explain a difference in activation of melanopsin-positive ipRGC by the angle of light exposure.

The pupil filters the amount of light passing through the eye and that determines visual acuity and detection performance [26, 27]. Studies have been reported that the melatonin suppression in individuals with pharmacological pupil dilation and the correlation between pupil diameter and melatonin suppression under the normal ceiling light [28, 29], but no study has been investigated the angle of light exposure, pupillary light reflex, and melatonin suppression. In the human retina, the distribution of melanopsin-positive ipRGC is most predominant in the central retina [30] and several studies have partly indicated the pupillary size may not affect the photosensitivity of central retina in human while using Ganzfeld light [31, 32]. Thus, the lower angle light exposure (light at 28°) may still grant luminous flux entered in central retina to adequately suppress nocturnal melatonin production nonetheless the diameter of pupil size is smaller than the higher light angle (Fig. 4).

Although we have a significant effect in the angle of incident light, we had limited controls on the prior light history and individual differences in the retinal photosensitivity which might affect the degree of nocturnal melatonin suppression [33]. In addition, vitreous body and permeability of lens [34] and lens transmission [35] decrease with aging which might also influenced by the present results. Additional studies may be necessary for us to deeper understand of our results. It is also interesting to further investigate whether the effect of light incident angle modulates circadian rhythms and sleep/wake cycle in human behavior.

## Conclusion

The present study showed that the angle of light incidence influence the nocturnal melatonin production. It suggests the light incident angle is an important factor for home and/or clinical use of head-mounted light therapy devices.

## Data Availability

The data used in the present study are available from the corresponding author upon reasonable request.
